# Cardioneuroablation versus cardiac pacemaker in the management of dominant cardioinhibitory vasovagal syncope: a retrospective cohort study

**DOI:** 10.1007/s10840-026-02327-5

**Published:** 2026-04-20

**Authors:** Alexander Álvarez-Ortiz, Leonor Eugenia Mariño-Murillo, Juan Carlos Zerpa, Jose P. Lopez-Lopez, María Alejandra Álvarez-Mariño, Tolga Aksu

**Affiliations:** 1 Instituto del Corazón de Bucaramanga, Bucaramanga, Colombia; 2 Masira Research Institute, Universidad de Santander (UDES), Bucaramanga, Colombia; 3 Electrophysiology, Pacemaker and Arrhythmias Service, HCor- Hospital do Coração, São Paulo, Brazil; 4 Universidad de Santander (UDES), Bucaramanga, Colombia; 5 Department of Cardiology, Istinye University, Medical Park Florya Hospital, Istanbul, 34742 Türkiye

**Keywords:** Cardioneuroablation, Neurocardiogenic syncope, Dual-chamber pacemaker with closed-loop stimulation (MP-CLS)

## Abstract

**Background:**

Refractory dominant cardioinhibitory vasovagal syncope (VVS) remains a therapeutic challenge in patients older than 40 years. Dual-chamber pacemakers with closed-loop stimulation (CLS-PM) are guideline-supported, while cardioneuroablation (CNA), targeting ganglionated plexus, has emerged as a device-free alternative. Direct comparative data between these strategies are limited.

**Methods:**

We conducted a retrospective, comparative cohort study including patients > 40 years with refractory cardioinhibitory VVS treated between 2018 and 2024. Patients underwent either CNA with extracardiac vagal stimulation validation (CNA-ECVS) or CLS-PM implantation. The primary outcome was recurrence of the first syncope episode. Secondary outcomes included syncope-free survival, syncope burden (≥ 50% reduction), and safety. Kaplan–Meier analysis and Cox proportional hazards models were applied.

**Results:**

Eighty patients were included (37 CNA-ECVS, 43 CLS-PM; mean age 58.0 ± 14.6 years). Patients in the CNA group were younger than those in the CLS-PM group (49.9 ± 10.3 vs. 65.0 ± 14.1 years; *p* < 0.001). During a mean follow-up of 3.8 years, syncope-free rates were 89.2% in the CNA group and 79.1% in the CLS-PM group (*p* = 0.363). Syncope-free survival did not differ significantly (log-rank *p* = 0.168), and recurrence risk was similar between strategies (HR 0.45; 95% CI 0.14–1.46; *p* = 0.182). Post-procedural syncope burden was numerically lower after CNA (10.8% vs. 20.9%) but without statistical significance. No major adverse events were observed in either group.

**Conclusions:**

In patients older than 40 years with refractory cardioinhibitory NCS, CNA with physiological validation and CLS-PM therapy demonstrated comparable efficacy and safety. Both strategies represent viable therapeutic options, warranting confirmation in randomized clinical trials.

**Graphical abstract:**

**Figure:**
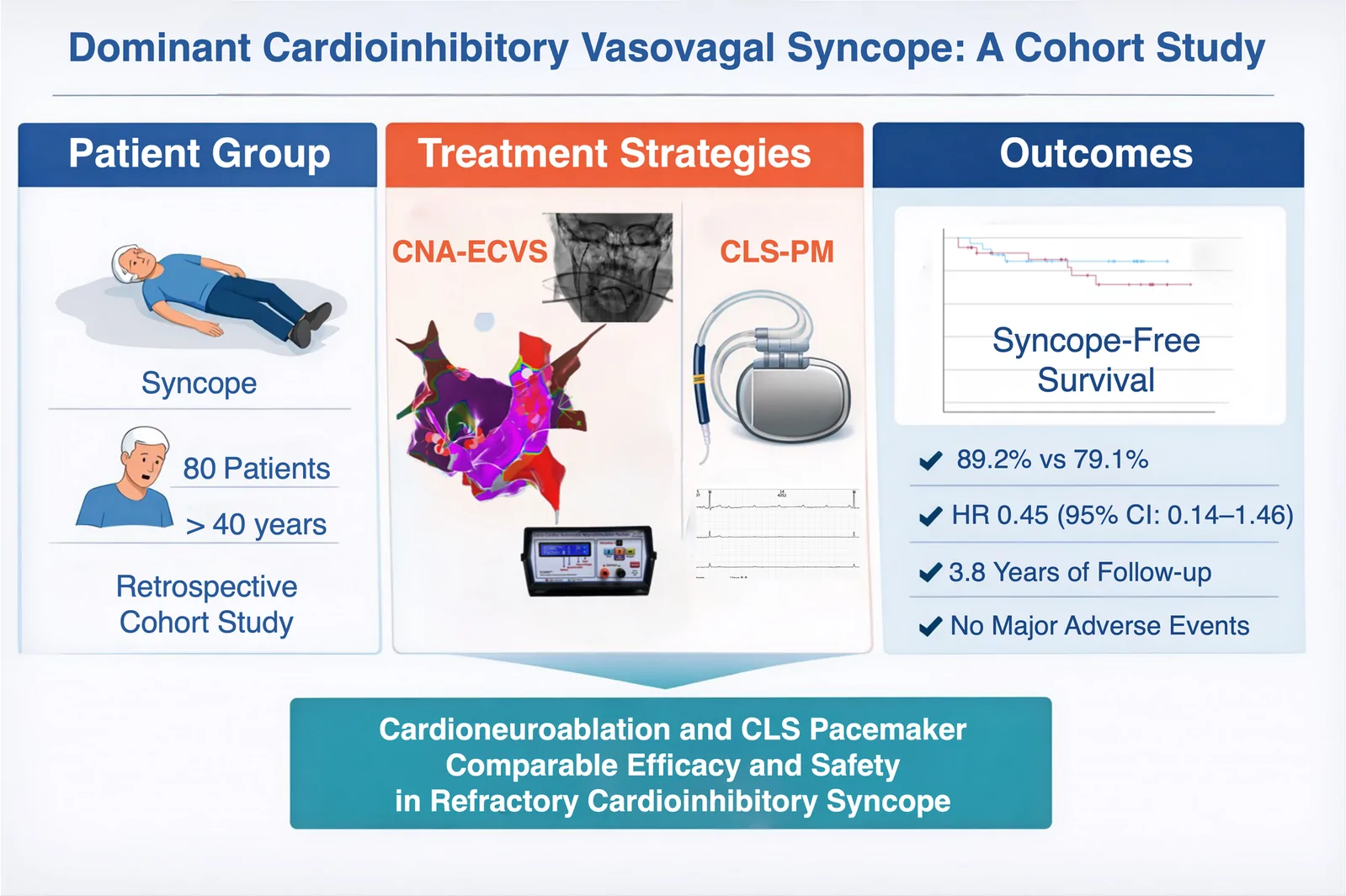

## Introduction

Vasovagal syncope (VVS) is the most common cause of reflex syncope [1, 2]. Although the exact mechanism has not been fully elucidated, it is widely accepted that the combination of sympathetic inhibition and parasympathetic hyperactivity can lead to hypotension (vasodepressor component) and/or bradycardia/asystole (cardioinhibitory component), resulting in transient loss of consciousness due to global cerebral hypoperfusion [1, 2].

While most cases occur in an isolated or situational manner, a subgroup of patients develops recurrent and incapacitating episodes, leading to functional impairment and a significant healthcare burden [1]. In this setting, non-pharmacological measures—including patient education regarding diagnosis and prognosis, avoidance of triggering factors, increased salt and water intake, and physical counterpressure maneuvers—as well as pharmacological therapy, may be beneficial; however, these strategies do not always achieve adequate control in severe and refractory forms of the disease [1–5]. Pathophysiological classification (vasodepressor, cardioinhibitory, or mixed) is crucial to guide therapeutic decisions, as the magnitude of the vasodepressor component may limit the effectiveness of interventions focused on heart rate control; therefore, tilt-table testing remains an important tool for phenotyping the hemodynamic response [6, 7].

Permanent cardiac pacing has been shown to reduce recurrence in patients with severe and recurrent VVS with a predominant cardioinhibitory component, particularly in that refractory to conservative and/or pharmacological measures [8–10]. According to the guidelines, dual-chamber pacemaker implantation should be considered in patients older than 40 years with refractory cardioinhibitory type VVS [1, 2].

In parallel, cardioneuroablation (CNA) has emerged as a strategy aimed at modulating autonomic control through endocardial radiofrequency ablation of cardiac ganglionated plexus (GP) [11–21]. The goal of CNA is to attenuate the excessive vagal discharge responsible for cardioinhibition. In selected populations, CNA has been associated with low rates of syncope recurrence. Recent meta-analyses demonstrated that among individuals with cardioinhibitory VVS treated with CNA, the pooled prevalence of syncope recurrence was lower than 10% [16, 22].

Despite these advances, uncertainty remains as to whether CNA or pacemaker therapy provides superior outcomes in patients with refractory cardioinhibitory VVS. A multicenter retrospective study comparing CNA with permanent pacemaker therapy reported a similar safety profile and inconclusive differences in recurrence between both strategies [17].

Therefore, the present study evaluated, in a retrospective cohort of adults older than 40 years with refractory cardioinhibitory neurocardiogenic syncope, the effect of CNA with extracardiac validation compared with dual-chamber closed-loop stimulation pacemaker therapy (CLS-PM) on first syncope recurrence, event-free survival, syncope burden, and safety outcomes.

## Methods

### Study design

A retrospective, comparative observational cohort study was conducted at a single high-volume tertiary center. Patients treated between January 1, 2018, and December 31, 2024, were included. CNA with extracardiac vagal stimulation (CNA-ECVS) and CLS-PM were compared as treatment strategies for refractory cardioinhibitory neurocardiogenic syncope.

### Study population and inclusion criteria

Individuals older than 40 years with dominant cardioinhibitory VVS and ≥ 2 syncope episodes within the previous 12 months were included, despite non-pharmacological measures (identification and avoidance of vasovagal triggers, adequate hydration with increased salt and water intake, and training in physical counterpressure maneuvers such as handgrip and leg crossing) and pharmacological therapy (e.g., fludrocortisone and/or midodrine).

The diagnosis of a cardioinhibitory phenotype was established after systematic clinical evaluation and exclusion of alternative causes, with documentation of syncope-associated asystole during available monitoring and/or a positive cardioinhibitory response on tilt-table testing (including episodes of asystole during the study).

Exclusion criteria included age ≤ 40 years; syncope attributable to non-reflex causes (e.g., primary arrhythmias, significant structural heart disease, or other neurological/metabolic etiologies) based on the documented clinical evaluation; and insufficient evidence to support a cardioinhibitory phenotype. Additionally, patients with incomplete clinical records or inadequate follow-up precluding reliable outcome assessment were excluded. When available, significant left ventricular systolic dysfunction (e.g., left ventricular ejection fraction < 40%) was also considered an exclusion criterion due to its potential prognostic and therapeutic confounding effect.

Participants were required to have received one of the two evaluated strategies—CNA-ECVS or CLS-PM—and to have sufficient information available to assess outcomes (recurrence and time to recurrence, as well as adverse events).

### Cardioneuroablation procedure

CNA was performed according to classical descriptions of the technique, under general anesthesia with orotracheal intubation [15]. Parasympatholytic agents were avoided throughout the procedure. During ECVS, neuromuscular blockade was administered to minimize patient movement and reduce the risk of cervical injury.

Three-dimensional electroanatomical mapping was performed using the CARTO 3 system (Biosense Webster, Irvine, CA, USA) to guide the procedure. Ablation was carried out using an open-irrigated 3.5-mm tip contact force–sensing catheter (THERMOCOOL SMARTTOUCH™, Biosense Webster), connected to a radiofrequency generator operating in power-controlled mode. Saline irrigation was applied according to the manufacturer’s recommendations for atrial procedures.

**Fig. 1:**
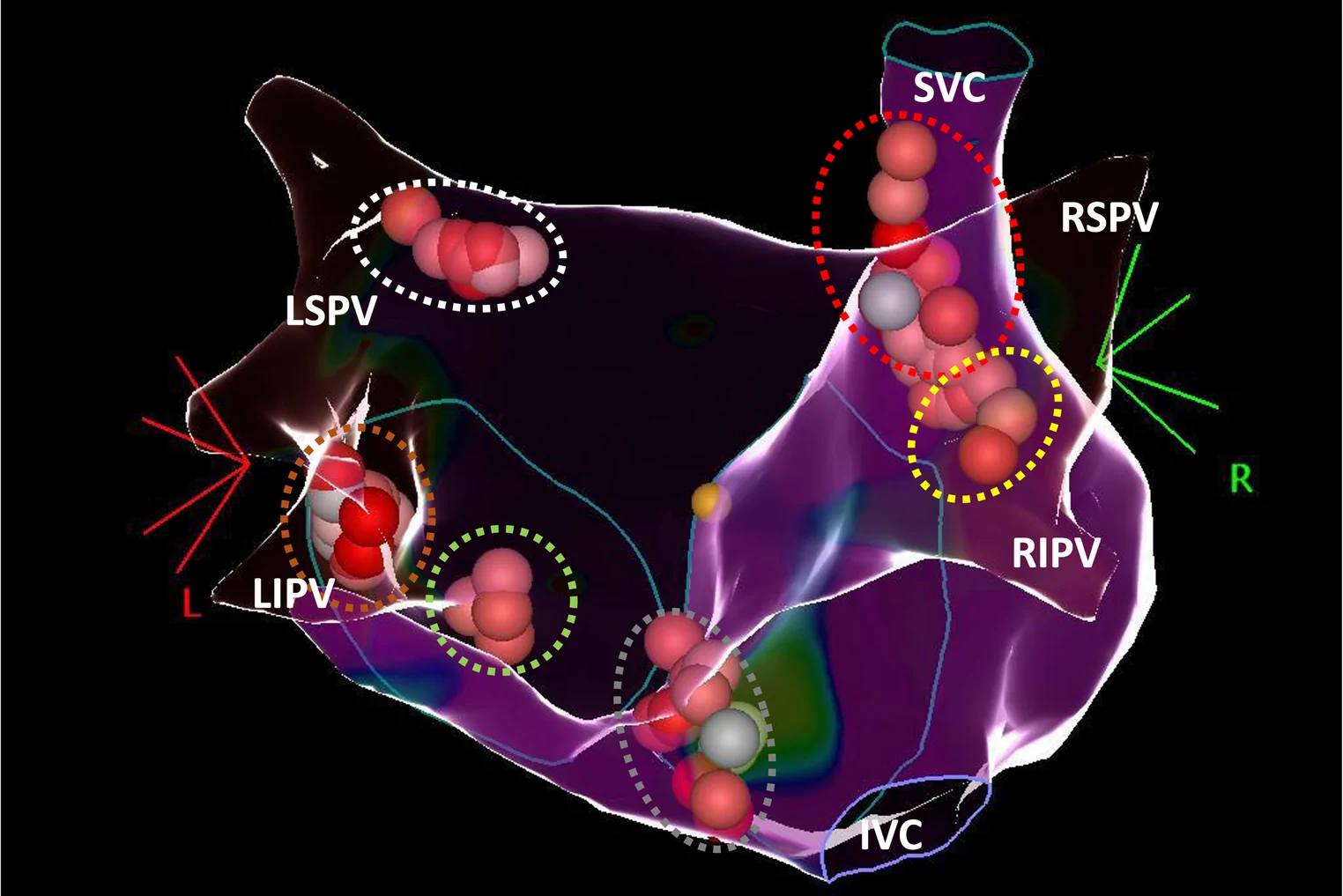
Three-dimensional electroanatomical map of the left and right atria showing the distribution of ablation points during cardioneuroablation. Clusters of lesions are identified over anatomical regions corresponding to the main cardiac ganglionated plexi (left superior ganglionated plexus-white circle, left inferior ganglionated plexus-green circle, left posteromedial ganglionated plexus-gray circle, right superior ganglionated plexus-red circle, and right inferior ganglionated plexus-yellow circle), with the aim of achieving autonomic modulation. Sites where autonomic potentials and fractionated electrograms were recorded are also indicated, which guided lesion delivery in the cardioneuroablation target areas. IVC, inferior vena cava; LIPV, left inferior pulmonary vein; LSPV, left superior pulmonary vein; RIPV, right inferior pulmonary vein; RSPV, right superior pulmonary vein, SVC, superior vena cava

Target regions were determined by integrating biatrial three-dimensional reconstruction with the anatomical localization of the main cardiac ganglionated plexi as previously described in the literature (Fig. 1). In the left atrium, the following regions were targeted: (a) the left superior ganglionated plexus, located in the antral region of the left superior pulmonary vein along the ridge between this vein and the left atrial appendage; (b) the left inferolateral ganglionated plexus, situated in the inferolateral/antral region of the left pulmonary veins adjacent to the posteroinferior atrial wall; and (c) the left posteromedial ganglionated plexus, located in the posteromedial left atrium near the posterior mitral annulus and the left inferior pulmonary vein. In the right atrium, the following regions were addressed: (a) the right superior paraseptal ganglionated plexus, located around the junction of the superior vena cava and the right atrium, in close anatomical proximity to the sinus node; and (b) the right inferior paraseptal ganglionated plexus, located in the posteroinferior right atrium near the junction of the inferior vena cava with the right atrium and the ostium of the coronary sinus.

Energy settings followed standard atrial ablation practice, with power delivery of 30–40 W in both the left and right atria, a maximum temperature limit of 45 °C, and an irrigation flow rate of 17–25 mL/min. Each radiofrequency application was delivered for 30–60 s or until resolution of fractionated electrograms at the target site with a clear reduction in local bipolar voltage. Lesions were applied using a point-by-point strategy over the predefined ganglionated plexus regions. Additional applications were delivered when fractionated potentials persisted or when extracardiac vagal stimulation continued to elicit a clinically relevant vagal response.

Acute procedural efficacy was defined by a composite endpoint that included (a) elimination of fractionated electrograms at the treated ganglionated plexus regions and (b) abolition of the vagal response (bradycardia, atrioventricular block, or asystole) during repeat ECVS at the end of the procedure. After confirmation of the absence of a vagal response on final extracardiac stimulation, intravenous atropine (2–3 mg) was administered as an ancillary physiological check. Given the ECVS-confirmed denervation, no clinically meaningful chronotropic response is expected after atropine (only minimal heart rate variation may occur). Adequate vagal denervation was defined by the simultaneous presence of (a) absence of vagal response on final ECVS and (b) elimination of fractionated electrograms at target sites; atropine testing was considered supportive but not a standalone determinant of acute success.

All CNA procedures and extracardiac vagal stimulation validations were performed by a single experienced electrophysiologist specialized in complex ablation, following a standardized protocol for mapping, ganglionated plexus identification, and physiological confirmation of effect using extracardiac vagal stimulation.

### Extracardiac vagal stimulation (ECVS)

**Fig. 2:**
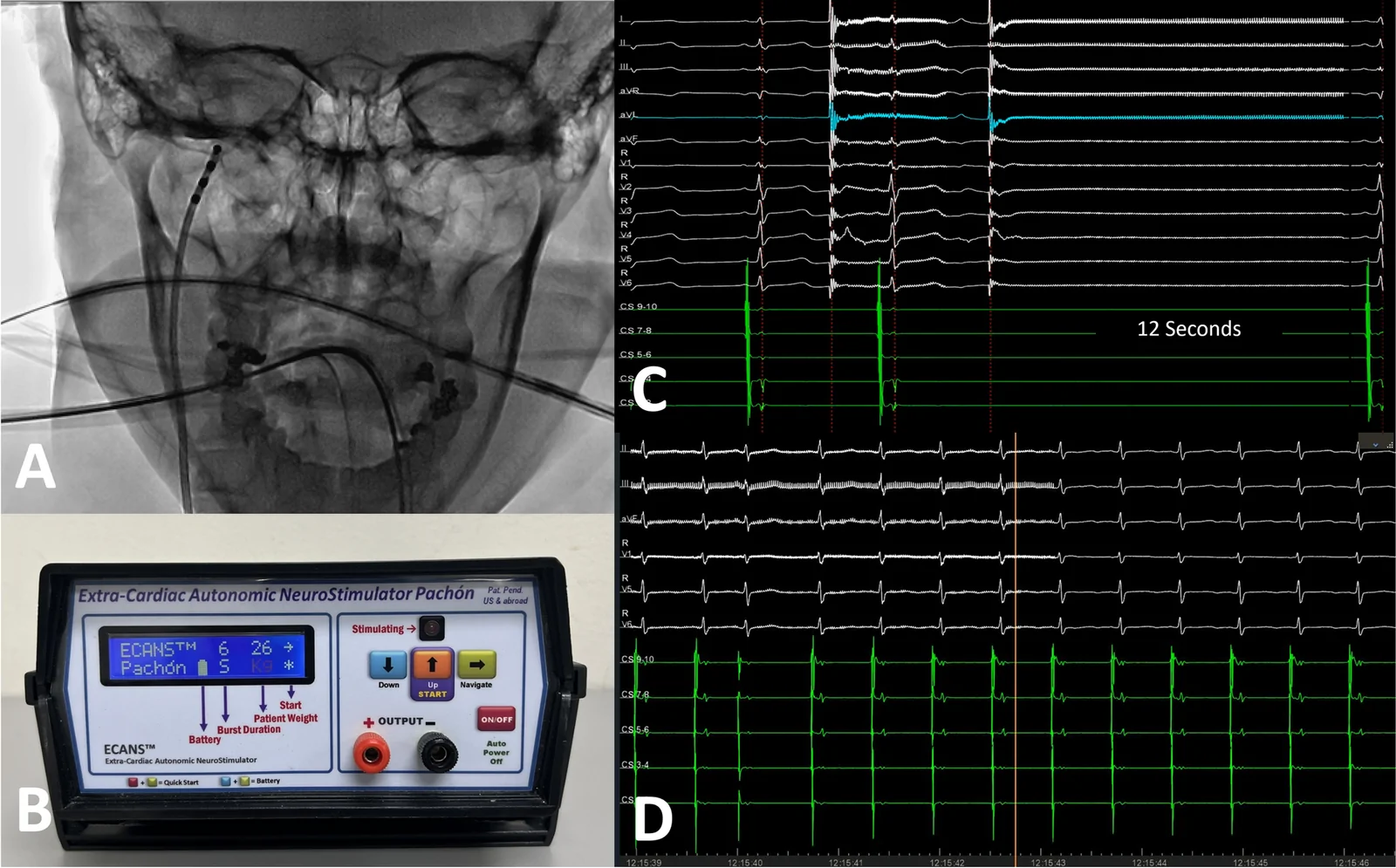
(**A**) Fluoroscopic image demonstrating the position of the quadripolar catheter introduced through the right internal jugular vein and positioned at the level of the jugular foramen, used for extracardiac vagal stimulation. (**B**) Programmable neurostimulation device for extracardiac vagal stimulation (Extracardiac Autonomic Nerve Stimulator, ECANS, Pachón; São Paulo, Brazil) used before and after the cardioneuroablation procedure, illustrating the adjustable parameters of stimulation frequency, pulse duration, and output amplitude. (**C**) Extracardiac vagal stimulation using a neurostimulator before cardioneuroablation, demonstrating a 12-second episode of asystole. (**D**) Extracardiac vagal stimulation using a neurostimulator after cardioneuroablation, showing absence of pause or atrioventricular conduction block

A quadripolar catheter was advanced through the right and left internal jugular veins and positioned at the level of the jugular foramen, in close anatomical proximity to the vagus nerve, under fluoroscopic guidance using anteroposterior, right anterior oblique 20°, and left anterior oblique 30° projections (Fig. 2A). When necessary, contrast venography was performed to confirm the cranial position of the catheter.

ECVS was delivered through the distal electrode pair using a programmable neurostimulator (Extracardiac Autonomic Nerve Stimulator, ECANS, Pachón; São Paulo, Brazil) (Fig. 2B). Trains of electrical pulses were applied at 50 Hz, with a pulse width of 50 µs and a duration of 5 s. Output amplitude was adjusted to 1 V/kg of body weight, up to a maximum of 70 V, or until reproducible induction of transient asystole or high-grade atrioventricular (AV) block was achieved.

Simultaneously, atrial pacing was performed from a quadripolar catheter positioned along the lateral wall of the right atrium to ensure atrial capture and to confirm the vagal response pattern. ECVS was systematically performed before ablation to document baseline vagal response, during the procedure to guide the progression of denervation, and at the end of the intervention to verify complete abolition of the vagal response (Fig. 2C, D).

### Dual-chamber pacemaker implantation and programming with closed-loop stimulation (CLS-PM)

After patient inclusion, a transvenous dual-chamber pacemaker capable of programming in DDD mode with a Closed-Loop Stimulation (DDD-CLS) algorithm was implanted (e.g., Biotronik platforms such as Evia, Eluna, and Edora). Device implantation was performed using a standard technique via ultrasound-guided subclavian or cephalic venous access, with atrial and ventricular lead positioning under fluoroscopic guidance and intraoperative verification of appropriate electrical parameters.

Subsequently, devices were programmed in DDD-CLS mode with the following settings: minimum pacing rate (day/night) of 50 bpm, maximum pacing rate of 160 bpm, CLS rate of 110 bpm, with Dynamic CLS set to “high” and the dynamic rate limit set to “off.” The atrioventricular (AV) interval was programmed at 250–180 milliseconds (ms), with AV hysteresis set at 400 ms; the atrial refractory period was programmed at 400 ms. Both pacing and sensing polarities were configured as bipolar. Finally, baseline pacing outputs immediately after implantation (atrial and ventricular) were programmed at 3.5 V with a pulse width of 0.4 ms. These outputs were subsequently adjusted automatically through periodic autocapture algorithms in each chamber to ensure an adequate safety margin.

### Outcomes

The primary efficacy outcome was syncope-free survival after the first post-procedural syncope episode, assessed over the available clinical follow-up. Secondary outcomes included the proportion of patients who remained syncope-free at the end of follow-up, recurrence-free time (defined as the interval from the procedure to the first event), and post-intervention syncope burden, expressed both as the proportion of patients with recurrence and as clinical response defined by a ≥ 50% reduction in the number of episodes compared with the baseline period. In addition, procedure- or device-related adverse events were recorded in both groups.

### Statistical analysis

Statistical analyses were performed using IBM SPSS Statistics (version 30.0). Continuous variables were summarized as mean ± standard deviation or as median (interquartile range), according to their distribution, and categorical variables were expressed as frequencies and percentages. Comparisons between groups (CNA-ECVS vs. CLS-PM) were conducted using the chi-square test or Fisher’s exact test for categorical variables, and the Mann–Whitney U test for continuous variables.

Time-to-event outcomes (recurrence of the first syncope episode) were analyzed using the Kaplan–Meier method, with between-group comparisons performed using the log-rank (Mantel–Cox) test. Additionally, the association between intervention type and recurrence was estimated using a Cox proportional hazards model, with results reported as hazard ratios (HRs) and 95% confidence intervals (95% CI). The model was adjusted for prespecified clinical covariates, including age, sex, and syncope frequency during the 12 months prior to the procedure. A two-sided *p* value < 0.05 was considered statistically significant.

### Ethical Approval and Confidentiality Considerations

This retrospective, comparative observational cohort study was reviewed and approved by the Institutional Ethics Committee of the Instituto del Corazón de Bucaramanga (Approval No. 003/004-08-2023). The research was conducted in accordance with ethical principles for research involving human subjects and applicable national regulations, in compliance with the Declaration of Helsinki and its subsequent amendments. Given the retrospective nature of the study, data were obtained from pre-existing medical records and institutional databases, and all information was anonymized/coded prior to analysis to ensure patient confidentiality. In this context, and as determined by the ethics committee, a waiver of individual informed consent was granted, as the study involved secondary data only and did not entail additional interventions or modifications to routine clinical care.

## Results

### Baseline characteristics

A total of 80 patients with cardioinhibitory neurocardiogenic syncope were included (Table 1), of whom 37 (46.25%) were treated with CNA-ECVS and 43 (53.75%) with CLS-PM. The mean age of the cohort was 58.01 ± 14.55 years, with patients in the CNA group being younger than those in the CLS-PM group (49.86 ± 10.32 vs. 65.02 ± 14.08 years; *p* < 0.001). Overall, 36.2% of participants were male (*n* = 29), with a higher proportion of men in the CLS-PM group compared with the CNA-ECVS group (48.8% vs. 21.6%; *p* = 0.012).

**Table 1:** Baseline Characteristics of the Patients

Variable	Total (*n* = 80), n (%) / Mean ± SD	CNA (*n* = 37), n (%) / Mean ± SD	CLS-PM (*n* = 43), n (%) / Mean ± SD	*p* value
Age (years)	58.01 ± 14.55	49.86 ± 10.32	65.02 ± 14.08	< 0.001
Male sex	29 (36.2)	8 (21.6)	21 (48.8)	0.012
Asystole on tilt-table test	41 (51.2)	23 (62.2)	18 (41.9)	0.070
Total number of syncope episodes	7.10 ± 3.15	7.19 ± 3.13	7.02 ± 3.20	0.314
Syncope episodes in the last 12 months	3.00 ± 1.79	2.89 ± 1.90	3.09 ± 1.70	0.144
Orthostatic hypotension	7 (8.8)	3 (8.1)	4 (9.3)	0.98
Fludrocortisone use	13 (16.2)	9 (24.3)	4 (9.3)	0.069
Midodrine use	24 (30.0)	15 (40.5)	9 (20.9)	0.056
Antidepressant use	14 (17.5)	12 (32.4)	2 (4.7)	0.001
Diabetes mellitus	14 (17.5)	5 (13.5)	9 (20.9)	0.384
Hypertension	27 (33.8)	9 (24.3)	18 (41.9)	0.098
Left ventricular ejection fraction (%)	60.65 ± 3.74	61.22 ± 3.38	60.16 ± 3.99	0.126
Weight (kg)	71.20 ± 11.12	70.97 ± 11.15	71.40 ± 11.21	0.728
Height (cm)	161.53 ± 8.17	158.70 ± 7.30	163.95 ± 8.17	0.008
Body mass index (kg/m²)	27.16 ± 4.11	28.21 ± 3.60	26.25 ± 4.34	< 0.001
Systolic blood pressure (mmHg)	133.66 ± 19.03	133.97 ± 18.04	133.40 ± 20.04	0.896
Diastolic blood pressure (mmHg)	73.35 ± 10.76	75.03 ± 8.69	71.91 ± 12.19	0.081

*p* values were calculated using the chi-square test or Fisher’s exact test for categorical variables, and the Mann–Whitney U test for quantitative variables. Quantitative data are presented as mean ± SD (standard deviation)

Asystole during tilt-table testing was documented in 51.2% of participants (*n* = 41), occurring more frequently in the CNA-ECVS group than in the CLS-PM group (62.2% vs. 41.9%), although this difference did not reach statistical significance (*p* = 0.070). Mean baseline syncope burden was 7.10 ± 3.15, with similar values between groups (CNA-ECVS 7.19 ± 3.13 vs. CLS-PM 7.02 ± 3.20; *p* = 0.314). Likewise, the mean number of syncope episodes in the 12 months preceding the procedure was 3.00 ± 1.79, with no significant differences between the CNA-ECVS and CLS-PM groups (2.89 ± 1.90 vs. 3.09 ± 1.70; *p* = 0.144). Orthostatic hypotension was reported in 7 patients (8.8%), with comparable proportions in the CNA-ECVS (8.1%) and CLS-PM (9.3%; *p* = 0.98) groups.

Regarding pharmacological treatments, fludrocortisone use was observed in 16.2% of the overall cohort, without significant differences between the CNA-ECVS and CLS-PM groups (24.3% vs. 9.3%; *p* = 0.069). Similarly, midodrine use was reported in 30.0% of patients, with a higher proportion in the CNA-ECVS group compared with the CLS-PM group (40.5% vs. 20.9%; *p* = 0.056), although this difference was not statistically significant. Antidepressant use was significantly more frequent in the CNA-ECVS group than in the CLS-PM group (32.4% vs. 4.7%; *p* = 0.001). No significant differences were observed between groups in the prevalence of diabetes mellitus (17.5% overall; *p* = 0.384) or hypertension (33.8% overall; *p* = 0.098). Left ventricular ejection fraction was preserved in both groups, with an overall mean of 60.65 ± 3.74%, and no significant differences between CNA-ECVS and CLS-PM (61.22 ± 3.38 vs. 60.16 ± 3.99; *p* = 0.126). The remaining anthropometric and vital sign variables are presented in Table 1.

### Syncope recurrence-free survival

During a mean follow-up of 3.8 years, 89.2% of patients in the CNA group (33/37) and 79.1% of those in the CLS-PM group (34/43) remained free of recurrence. Mean recurrence-free survival, estimated using the Kaplan–Meier method, was comparable between both treatment strategies: 194.6 days (95% CI: 175.8–213.5) for CNA and 194.7 days (95% CI: 169.2–220.2) for CLS-PM, with no significant differences between survival curves (log-rank *p* = 0.168) (Table 2).

**Table 2:** Syncope Recurrence-Free Survival According to Therapeutic Strategy (CNA vs. CLS-PM)

Procedure	Mean (days)	95% CI (mean)	Patients with Events (*n*)	Patients without Events (*n*)	Event-free (%)
Cardioneuroablation	194.6 ± 9.6	175.8–213.5	4	33	89.2%
CLS Pacemaker	194.7 ± 13.0	169.2–220.2	9	34	79.1%

The *p* value for the log-rank (Mantel–Cox) test was 0.168

### Reduction in syncope burden

In the assessment of post-procedural syncope burden, syncope recurrence was recorded in 4 of 37 patients (10.8%) treated with CNA-ECVS and in 9 of 43 patients (20.9%) treated with CLS pacemaker therapy. Accordingly, the proportion of syncope-free patients was 89.2% in the CNA group and 79.1% in the CLS-PM group. Although an absolute reduction of 10.1% in recurrence was observed in favor of CNA, this difference did not reach statistical significance (chi-square *p* = 0.221; Fisher’s exact test *p* = 0.363) (Table 3).

**Table 3:** Reduction in Syncope Burden After the Procedure: CNA versus CLS-PM

Procedure	Total (*N*)	Recurrence (*n*) (Events)	No Recurrence (*n*) (Events)	With Syncope (%)	Syncope-Free (%)
Cardioneuroablation	37	4	33	10.8%	89.2%
CLS Pacemaker	43	9	34	20.9%	79.1%

Comparison between proportions (CNA vs. CLS-PM): *p* = 0.221 (chi-square test); *p* = 0.363 (Fisher’s exact test)

### Post-procedural syncope-free survival

**Fig. 3:**
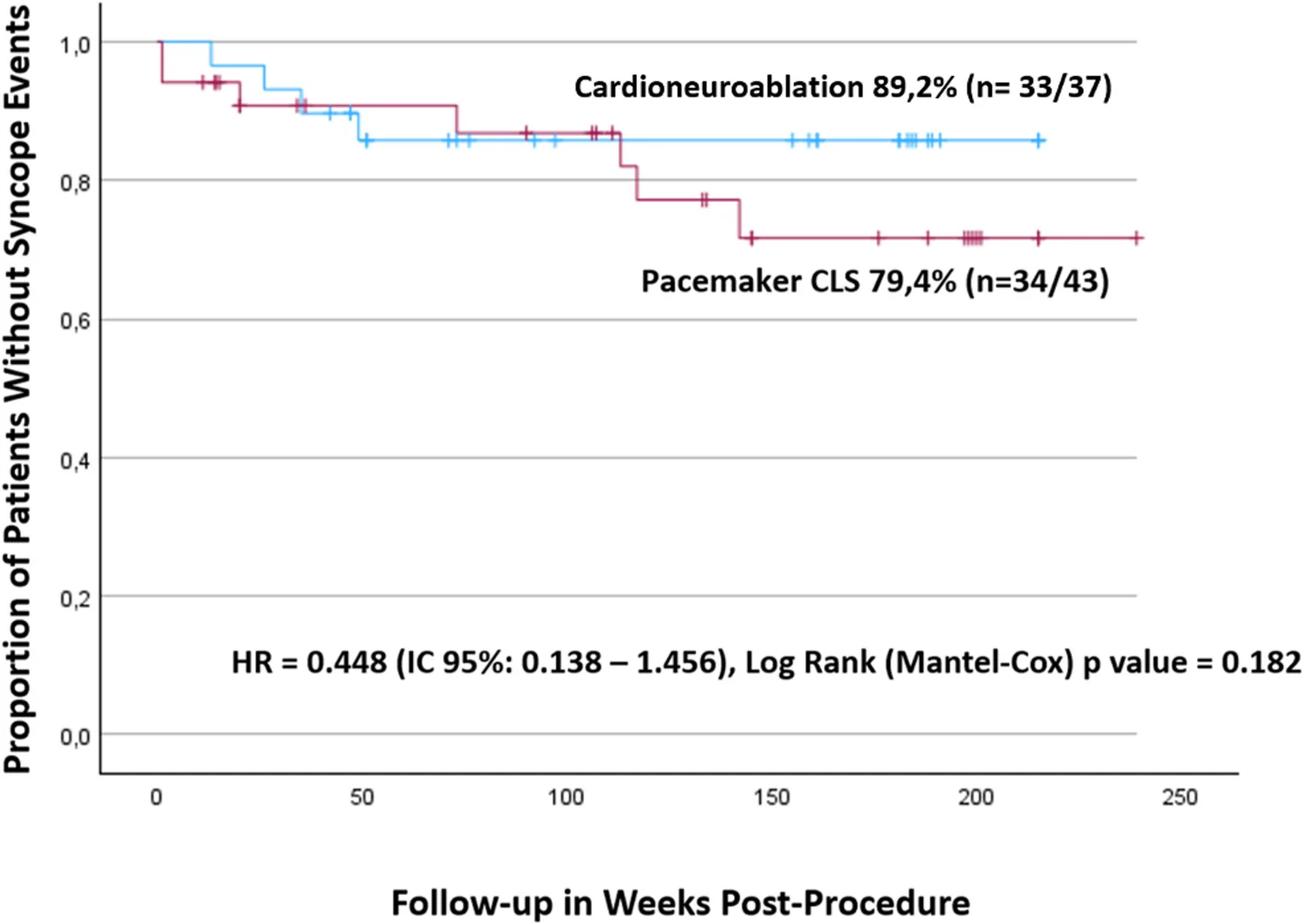
Syncope Recurrence-Free Survival After the Procedure (CNA vs. CLS-PM)

Figure 3 (Kaplan–Meier) depicts the time to recurrence of the first post-procedural syncope episode in both groups. Visually, the CNA and CLS-PM curves remain close and largely overlapping throughout most of the follow-up period, suggesting a broadly similar pattern of recurrence risk over time.

During follow-up, 4 events were recorded in the CNA group (4/37) and 9 events in the CLS-PM group (9/43), corresponding to syncope-free proportions of 89.2% and 79.1%, respectively. Comparison of survival curves did not demonstrate statistically significant differences (log-rank *p* = 0.168). In the Cox proportional hazards model, the type of procedure was not significantly associated with recurrence (HR = 0.448; 95% CI: 0.138–1.456; *p* = 0.182), with no evidence of superiority of either strategy.

### Safety profile

In this cohort, both treatment strategies demonstrated an overall favorable safety profile. No major complications were recorded, including pericardial effusion/tamponade, complete atrioventricular block, phrenic nerve palsy, cerebrovascular events (ischemic or hemorrhagic), or procedure-related mortality, as documented in the clinical records. In the CNA-ECVS group, two procedure-related complications were reported, consisting of one vascular injury and one case of deep vein thrombosis; no procedural complications of this type were observed in the CLS-PM group. No cases of pneumothorax or hemothorax were reported.

Additionally, given that a proportion of patients exhibited a concomitant vasodepressor component or required medical therapy (e.g., midodrine and/or fludrocortisone; Table 1), some patients continued or required adjunctive pharmacological treatment during follow-up for symptomatic control, without evidence of associated major complications. Overall, no increase in serious clinical outcomes attributable to either strategy was observed during the evaluated follow-up period.

### Validation by neurostimulation

In the group treated with cardioneuroablation (CNA; *n* = 37), extracardiac vagal stimulation (ECVS) was used as a functional tool to confirm, in real time, the effectiveness of vagal denervation during the procedure. Prior to ablation, ECVS reproducibly induced asystole or atrioventricular block, demonstrating the integrity of cardiac parasympathetic input. After completion of CNA, complete abolition of the vagal response was achieved in approximately 95% of cases (35/37 patients). In the remaining two patients, a residual vagal response persisted, prompting additional ablation applications targeting the relevant ganglionated plexi until suppression of the reflex was confirmed. These findings support the value of ECVS as a reproducible method for intraoperative validation of CNA efficacy (Fig. 2C-D).

## Discussion

The main findings of this study can be summarized as follows: (i) in patients older than 40 years with refractory cardioinhibitory VVS, both CNA-ECVS and CLS-PM appear to be reasonable clinical alternatives, as they showed similar outcomes in terms of recurrence and event-free survival in this cohort; (ii) ECVS provides an objective physiological criterion to confirm denervation and could be standardized as an intraoperative endpoint to improve reproducibility across centers; (iii) both approaches showed a favorable safety profile, with no major adverse events, although two procedure-related complications occurred in the CNA group (vascular injury and deep vein thrombosis).

From a pathophysiological perspective, the vasovagal reflex results from the interaction between parasympathetic activation and sympathetic inhibition, favoring bradycardia/asystole and/or vasodepression. In patients with a predominant cardioinhibitory phenotype, syncope may be recurrent and disabling. In this context, the 2017 ACC/AHA/HRS guidelines consider DDD pacing reasonable in selected patients aged ≥ 40 years with recurrent episodes and prolonged spontaneous pauses [2]. Similarly, the 2018 ESC guidelines provide a comprehensive framework for the evaluation and management of reflex syncope, emphasizing clinical stratification and the use of tests such as tilt-table testing to define the hemodynamic phenotype [1]. Randomized evidence has supported pacing strategies in patients with documented cardioinhibition, including the ISSUE-3 trial and related studies, particularly when significant asystole is documented during symptomatic episodes or tilt testing [8–10].

Moreover, the efficacy of the CLS algorithm in reducing recurrence has been consistently demonstrated compared with alternative pacing strategies (CLS-off, “sham” DDI, or DDD with rate drop response), with robust evidence from the SPAIN trial and subsequent studies in severe reflex syncope [8]. In parallel, observational studies have suggested that leadless pacing may offer efficacy comparable to transvenous dual-chamber pacing (with CLS or rate drop response) in refractory cardioinhibitory VVS, although without proven superiority and with inherent limitations of non-randomized designs [23–25]. This body of evidence supports the role of CLS-PM as an appropriate and demanding “active comparator” when evaluating alternative strategies such as CNA. Management of these patients should therefore be considered within the spectrum of available therapies, including conservative measures, pharmacological treatment, and careful selection of candidates for advanced interventions. Trials such as POST and POST-IV, as well as studies evaluating selective serotonin reuptake inhibitors in refractory syncope, highlight the heterogeneity of responses to medical therapies and the need for individualized management (3–5).

Conversely, CNA represents a physiologically targeted approach aimed at attenuating the cardioinhibitory component through functional parasympathetic denervation achieved by ablation of GP [11–21]. The benefit of CNA was first described by Pachón et al. [13] in 2005 and subsequently supported by series reporting high acute success rates and syncope-free survival approaching 90% at mid-term follow-up [14]. Comparative studies versus conservative therapy have demonstrated substantial reductions in recurrence among patients with VASIS 2B or type 1 responses with asystole > 3 s [18]. Collectively, these data suggest that parasympathetic denervation through ganglionated plexus ablation is the most plausible mechanism underlying the observed benefit.

Direct comparisons between CNA and pacemaker therapy remain limited and are largely observational. In this regard, the multicenter retrospective study by Gopinathannair/Aksu and colleagues compared CNA with permanent pacing (including CLS, rate drop response, and leadless devices) and found similar efficacy in preventing recurrence and comparable safety profiles, emphasizing that both approaches may be reasonable in selected populations [17]. Our findings are consistent with this signal but add a more focused perspective by restricting the population to patients older than 40 years and using a contemporary comparator (CLS), which is clinically relevant because the discussion surrounding permanent pacing changes with age. In younger patients, concerns about long-term device-related complications remain substantial, whereas in patients older than 40 years, the risk–benefit balance is generally more favorable for pacing, in line with current guideline recommendations.

A distinctive and methodologically robust aspect of our series is the systematic use of ECVS as a physiological intraoperative validation tool. ECVS allows confirmation of intact vagal input before ablation and documentation of its abolition afterward, providing a functional endpoint that may be more reproducible than purely anatomical or electrogram-based approaches [26, 27]. In our cohort, complete abolition of the vagal response was achieved in approximately 95% of patients (35/37), supporting the robustness of this intraoperative endpoint and potentially reducing inter-operator variability. This approach aligns with the literature suggesting that more extensive strategies (left-sided or biatrial) are associated with better outcomes than right atrial–only ablation [16, 22, 28].

Regarding anatomical extent, more limited approaches—such as empirical ablation of the right superior ganglionated plexus to reduce the risks associated with transseptal access and left atrial ablation—have been proposed [19, 29]. However, aggregated evidence indicates that CNA confined to the right atrium may be associated with lower syncope-free survival compared with left-sided or biatrial approaches [16, 22]. This may be particularly relevant in patients with anatomical variability of GP or a more complex reflex pattern, in whom a biatrial strategy may provide more comprehensive modulation of the intrinsic cardiac autonomic nervous system.

With respect to safety, our study did not document major adverse events in either group, consistent with the available literature. Nevertheless, this finding should be interpreted cautiously. CNA is not free of procedural risks, including vascular complications, thrombosis, or pericardial effusion, and permanent pacing carries risks related to the device pocket, leads, venous access, and long-term device-related issues. In our cohort, two procedural complications occurred in the CNA group (vascular injury and deep vein thrombosis), without serious sequelae, and no cases of pneumothorax or hemothorax were observed. Additionally, inappropriate sinus tachycardia has been reported as a clinically relevant post-CNA event, potentially related to intrinsic heart rate variability or partial plexus ablation [30]. In our cohort, some patients required adjunctive therapy for a residual vasodepressor component (e.g., midodrine or fludrocortisone), which is consistent with the multifactorial nature of the vasovagal reflex.

Another debated issue is the potential proarrhythmic effect of ganglionated plexus ablation. Some reports have suggested possible atrial and ventricular proarrhythmic effects following ganglion ablation, although evidence remains heterogeneous [31–33]. In contrast, long-term evaluation of vagal denervation using Holter monitoring and heart rate variability has supported sustained autonomic changes after CNA, and other studies have suggested that CNA may not increase—and may even reduce—arrhythmic burden in selected cohorts [27]. These findings suggest that, at least with certain strategies and in specific populations, CNA does not necessarily increase arrhythmic risk.

The regional relevance of our study is noteworthy. To our knowledge, direct comparisons between CNA and a modern active comparator (CLS-PM) in a population restricted to patients older than 40 years with a cardioinhibitory phenotype are scarce, and most available evidence originates from Europe, North America, and Asia [34]. Our cohort therefore provides real-world data from South America. Moreover, our results are consistent with recent regional evidence: Álvarez Ortiz et al. [35] published in 2026 a combined retrospective–prospective cohort of 53 patients undergoing biatrial CNA with ECVS validation, reporting a recurrence rate of 13.2% and syncope-free survival of 86.8%, along with parasympathetic suppression (heart rate variability) and quality-of-life improvement, without major complications. This study provides external coherence supporting the effectiveness and safety of CNA with physiological validation in our setting and reinforces the plausibility of our comparative findings.

Finally, this study should be interpreted as hypothesis-generating. Its retrospective design, the limited number of events, and baseline differences between groups (age, sex, and adjunctive therapies) may introduce residual confounding, consistent with limitations described in prior observational studies. Nevertheless, our results suggest that in patients older than 40 years with refractory cardioinhibitory syncope, CNA-ECVS and CLS-PM may represent viable alternatives with similar efficacy and comparable safety. Multicenter randomized trials with longer follow-up are needed to more definitively determine which strategy offers greater net benefit, which GP should be targeted, and which mapping and validation tools should be standardized.

## Limitations

This study has several important limitations. First, its observational, retrospective, and non-randomized design precludes exclusion of selection bias and potential placebo effects, and limits causal inference. Comparisons between groups may have been influenced by baseline differences (e.g., older age and a higher proportion of men in the CLS-PM group, as well as variations in concomitant therapies), with the possibility of residual confounding despite Kaplan–Meier analysis and Cox modeling. Although we adjusted the time-to-event analysis for age, sex, and baseline syncope frequency, residual confounding related to non-random treatment allocation cannot be excluded.

Second, the total number of events was low and there was a high proportion of censored observations, which reduces statistical power to detect small or moderate differences between strategies. Additionally, outcome assessment relied on clinical records, with potential underreporting and heterogeneity in follow-up intensity. Standardized assessments of quality of life and longitudinal autonomic markers were not systematically incorporated across the entire cohort, limiting evaluation of patient-centered impact. Finally, follow-up duration may be insufficient to fully assess durability of effect and long-term phenomena such as parasympathetic reinnervation after GP ablation.

## Future directions

Based on the accumulated evidence and existing knowledge gaps, the following are needed:

- Multicenter randomized clinical trials comparing CNA versus CLS-PM therapy and/or conventional treatment in well-defined populations (e.g., patients older than 40 years with a cardioinhibitory phenotype).
- Long-term follow-up to evaluate recurrence, safety, and phenomena such as autonomic reinnervation and their clinical impact.
- Standardization of the technique, including definition of target sites, mapping strategies (e.g., AF-nests or other tools), and uniform criteria for acute success, incorporating physiological endpoints such as ECVS.
- Progressive integration of CNA into international recommendations and guidelines once robust comparative evidence becomes available to better define indications, patient selection, and clinical decision-making algorithms.

## Conclusions

In appropriately selected patients older than 40 years with refractory cardioinhibitory VVS, CNA-ECVS and CLS-PM represent viable therapeutic options with comparable safety profiles. Therapeutic decision-making should be individualized, taking into account baseline patient characteristics and the possible coexistence of a vasodepressor component, which may influence residual symptoms. Randomized trials with long-term follow-up are needed to more precisely define which patient subgroups derive greater benefit from each strategy.

## Data Availability

The datasets used and/or analyzed during the current study are available from the corresponding author upon reasonable request.

## References

[1] Brignole M, Moya A, de Lange FJ, Deharo JC, Elliott PM, Fanciulli A, Fedorowski A, Furlan R, Kenny RA, Martín A, Probst V, Reed MJ, Rice CP, Sutton R, Ungar A, van Dijk JG, ESC Scientific Document Group. 2018 ESC Guidelines for the diagnosis and management of syncope. Eur Heart J. 2018;39(21):1883–948. 29562304 10.1093/eurheartj/ehy037

[2] Shen WK, Sheldon RS, Benditt DG, Cohen MI, Forman DE, Goldberger ZD, Grubb BP, Hamdan MH, Krahn AD, Link MS, Olshansky B, Raj SR, Sandhu RK, Sorajja D, Sun BC, Yancy CW. 2017 ACC/AHA/HRS guideline for the evaluation and management of patients with syncope: executive summary: a report of the American College of Cardiology/American Heart Association Task Force on Clinical Practice Guidelines and the Heart Rhythm Society. J Am Coll Cardiol. 2017;70:620–63. 28286222 10.1016/j.jacc.2017.03.002

[3] Raj SR, Faris PD, McRae M. Rationale for the POST IV trial: assessment of midodrine. Clin Auton Res. 2012;22(6):275–80. 10.1007/s10286-012-0155-5. 22610268 PMC3439533

[4] Sheldon R, Connolly S, Rose S. Prevention of Syncope Trial (POST): a randomized study of metoprolol in vasovagal syncope. Circulation. 2006;113(9):1164–70. 10.1161/circulationAHA.105.594556. 16505178

[5] Di Girolamo E, Di Iorio C, Sabatini P, et al. Paroxetine hydrochloride for refractory vasovagal syncope: a randomized, double-blind, placebo-controlled study. J Am Coll Cardiol. 1999;33(5):1227–30. 10193720 10.1016/s0735-1097(98)00694-9

[6] Brignole M, Menozzi C, Del Rosso A, Costa S, Gaggioli G, Bottoni N, Bartoli P, Sutton R. New classification of haemodynamics of vasovagal syncope: beyond the VASIS classification. Analysis of the pre-syncopal phase of the tilt test without and with nitroglycerin challenge. Vasovagal Syncope Int Study Europace. 2000;2:66–76. 10.1053/eupc.1999.0064 11225598

[7] Sutton R, Kenny RA, Benditt DG. Syncope: Advances in Diagnosis and Treatment 2024. J Cardiovasc Electrophysiol. 2025;36(10):2671–6. 39736084 10.1111/jce.16546 PMC12530676

[8] Baron-Esquivias G, Morillo CA, Moya-Mitjans A, Martinez-Alday J, Ruiz-Granell R, Lacunza-Ruiz J, Garcia-Civera R, Gutierrez-Carretero E, Romero-Garrido R. Dual-Chamber Pacing With Closed Loop Stimulation in Recurrent Reflex Vasovagal Syncope: The SPAIN Study. J Am Coll Cardiol. 2017;70(14):1720–8. 28958328 10.1016/j.jacc.2017.08.026

[9] Brignole M, Menozzi C, Moya A, Andresen D, Blanc JJ, Krahn AD, Wieling W, Beiras X, Deharo JC, Russo V, Tomaino M, Sutton R. International Study on Syncope of Uncertain Etiology 3 (ISSUE-3) Investigators. Pacemaker therapy in patients with neurally mediated syncope and documented asystole: Third International Study on Syncope of Uncertain Etiology (ISSUE-3): a randomized trial. Circulation. 2012;125(21):2566–71. 22565936 10.1161/CIRCULATIONAHA.111.082313

[10] Brignole M, Russo V, Arabia F, Oliveira M, Pedrote A, Aerts A, Rapacciuolo A, Boveda S, Deharo JC, Maglia G, Nigro G, Giacopelli D, Gargaro A, Tomaino M. BioSync CLS trial Investigators. Cardiac pacing in severe recurrent reflex syncope and tilt-induced asystole. Eur Heart J. 2021;42(5):508–16. 33279955 10.1093/eurheartj/ehaa936 PMC7857694

[11] Aksu T, Guler TE, Bozyel S, Yalin K, Gopinathannair R. Usefulness of post-procedural heart rate response to predict syncope recurrence or positive head up tilt table testing after cardioneuroablation. Europace. 2020;22:1320–7. 32898255 10.1093/europace/euaa230

[12] Aksu T, Guler TE, Bozyel S, Golcuk SE, Yalin K, Lakkireddy D, Gopinathannair R. Medium-term results of Cardioneuroablation for clinical bradyarrhythmias and vasovagal syncope: effects on QT interval and heart rate. J Interv Card Electrophysiol. 2020;60(1):57–68. 32034611 10.1007/s10840-020-00704-2

[13] Pachon JC, Pachon EI, Pachon JC, Lobo TJ, Pachon MZ, Vargas RN, Jatene AD. Cardioneuroablation–new treatment for neurocardiogenic syncope, functional AV block and sinus dysfunction using catheter RF-ablation. Europace. 2005;7:1–13. 15670960 10.1016/j.eupc.2004.10.003

[14] Pachon JC, Pachon EI, Cunha Pachon MZ, Lobo TJ, Pachon JC, Santillana TG. Catheter ablation of severe neurally meditated reflex (neurocardiogenic or vasovagal) syncope: Cardioneuroablation long-term results. Europace. 2011;13:1231–42. 21712276 10.1093/europace/eur163

[15] Aksu T, Guler TE, Mutluer FO, Bozyel S, Golcuk SE, Yalin K. Electroanatomic-mapping-guided Cardioneuroablation versus combined approach for vasovagal syncope: a crosssectional observational study. J Interv Card Electrophysiol. 2019;54:177–88. 30054828 10.1007/s10840-018-0421-4

[16] Vandenberk B, Lei LY, Ballantyne B, Vickers D, Liang Z, Sheldon RS, Chew DS, Aksu T, Raj SR, Morillo CA. Cardioneuroablation for vasovagal syncope: a systematic review and meta-analysis. Heart Rhythm. 2022;S1547–5271(22):02088–94. 10.1016/j.hrthm.2022.06.017 35716859

[17] Gopinathannair R, Olshansky B, Turagam MK, Gautam S, Futyma P, Akella K, et al. Permanent pacing versus cardioneuroablation for cardioinhibitory vasovagal syncope. J Interv Card Electrophysiol. 2025;68:203–10. 36562915 10.1007/s10840-022-01456-x

[18] Aksu T, Padmanabhan D, Shenthar J, Yalin K, Gautam S, Valappil SP, Banavalikar B, Guler TE, Bozyel S, Tanboga IH, Lakkireddy D, Olshansky RB, Gopinathannair R. The benefit of Cardioneuroablation to reduce syncope recurrence in vasovagal syncope patients: a case-control study. J Interv Card Electrophysiol. 2021;63(1):77–86. 33527216 10.1007/s10840-020-00938-0

[19] Debruyne P, Rossenbacker T, Collienne C, Roosen J, Ector B, Janssens L, Charlier F, Vankelecom B, Dewilde W, Wijns W. Unifocal right-sided ablation treatment for neurally mediated syncope and functional sinus node dysfunction under computed tomographic guidance. Circ Arrhythm Electrophysiol. 2018;11(9):e006604. 30354289 10.1161/CIRCEP.118.006604

[20] Gigante C, Penela D, Viveros D, Falasconi G, Teresi L, Latini AC, Soto-Iglesias D, Franco-Ocaña P, Francia P, Alderete J, Turturiello D, Bellido AF, Zaraket F, Valeriano C, Mea R, Tonello B, Sanchez-Mollá L, De Lucia C, Matiello M, Fernández-Armenta J, San Antonio R, Saglietto A, Ortiz-Pérez JT, Martí-Almor J, Berruezo A. A tailored approach to cardioneuroablation for reflex syncope and functional bradycardia: results from the ELEGANCE multicentre study. Europace. 2026;28(1):euaf320. 41468149 10.1093/europace/euaf320 PMC12849814

[21] Aksu T, Brignole M, Calo L, Debruyne P, Di Biase L, Deharo JC, Fanciulli A, Fedorowski A, Kulakowski P, Morillo C, Moya A, Piotrowski R, Stec S, Sutton R, van Dijk JG, Wichterle D, Tse HF, Yao Y, Sheldon RS, Vaseghi M, Pachon JC, Scanavacca M, Meyer C, Amin R, Gupta D, Magnano M, Malik V, Schauerte P, Shen WK, Acosta JCZ. Cardioneuroablation for the treatment of reflex syncope and functional bradyarrhythmias: A Scientific Statement of the European Heart Rhythm Association (EHRA) of the ESC, the Heart Rhythm Society (HRS), the Asia Pacific Heart Rhythm Society (APHRS) and the Latin American Heart Rhythm Society (LAHRS). Europace. 2024;26(8):euae206. 39082698 10.1093/europace/euae206 PMC11350289

[22] Armani Prata A, Katsuyama E, Scardini P, Antunes V, Granja J, Coan AC, Fukunaga C. Pachón Mateos JC. Cardioneuroablation in patients with vasovagal syncope: An updated systematic review and meta-analysis. Heart Rhythm. 2025;22(2):526–35. 39067734 10.1016/j.hrthm.2024.07.103

[23] Gopinathannair R, Salgado BC, Olshansky B. Pacing for vasovagal syncope. Arrhythm Electrophysiol Rev. 2018;7:95–102. 29967681 10.15420/aer.2018.22.2 PMC6020179

[24] Turagam MK, Gopinathannair R, Park PH, Tummala RV, Vasamreddy C, Shah A, et al. Safety and efficacy of leadless pacemaker for cardioinhibitory vasovagal syncope. Heart Rhythm. 2020;17(9):1575–81. 32389681 10.1016/j.hrthm.2020.05.006

[25] Olshansky B. Vasovagal syncope: to pace or not to pace. J Am Coll Cardiol. 2017;70:1729–31. 28958329 10.1016/j.jacc.2017.08.025

[26] Pachon JC, Pachon EI, Pachon MZ, et al. Long-term outcomes of cardioneuroablation with and without extracardiac vagal stimulation confirmation in severe cardioinhibitory syncope. J Cardiovasc Electrophysiol. 2024;35(4):641–50. 38240356 10.1111/jce.16188

[27] Pachon JC, Pachon EI, Cunha MZ, et al. Long-term evaluation of vagal denervation by cardioneuroablation using Holter and HRV. Circ Arrhythm Electrophysiol. 2020;13(4):e008703. 33198486 10.1161/CIRCEP.120.008703

[28] Piotrowski R, Baran J, Sikorska A, Niedzwiedz M, Krynski T, Kulakowski P. Cardioneuroablation: comparison of acute effects of the right vs. left atrial approach in patients with reflex syncope: the ROMAN2 study. Europace. 2024;26(2):euae042. 38315895 10.1093/europace/euae042 PMC10873699

[29] Debruyne P, Rossenbacker T, Janssens L, et al. Durable physiological changes and decreased syncope burden after right-sided ablation in patients with reflex syncope. Circ Arrhythm Electrophysiol. 2021;14(6):e009272. 10.1161/CIRCEP.120.009747 PMC8208097 33999698

[30] Kulakowski P, Piotrowski R. Inappropriate Sinus Tachycardia Following Cardioneuroablation for Reflex Syncope: A Case Report and Review of the Literature Illustrating this Underappreciated Adverse Effect. Arrhythm Electrophysiol Rev. 2025;14:e12. 40607009 10.15420/aer.2025.01 PMC12215415

[31] Lo LW, Scherlag BJ, Chang HY, Lin YJ, Chen SA, Po SS. Paradoxical long-term proarrhythmic effects after ablating the head station ganglionated plexi of the vagal innervation to the heart. Heart Rhythm. 2013;10:751–7. 23357542 10.1016/j.hrthm.2013.01.030

[32] Osman F, Kundu S, Tuan J, Jeilan M, Stafford PJ, Ng GA. Ganglionic plexus ablation during pulmonary vein isolation—predisposing to ventricular arrhythmias? Indian Pacing Electrophysiol J. 2010;10:104–7. 20126597 PMC2811210

[33] Chung WH, Masuyama K, Challita R, Hayase J, Mori S, Cha S, Bradfield JS, Ardell JL, Shivkumar K, Ajijola OA. Ischemia-induced ventricular proarrhythmia and cardiovascular autonomic dysreflexia after cardioneuroablation. Heart Rhythm. 2023;20(11):1534–45. 37562487 10.1016/j.hrthm.2023.08.001 PMC13195725

[34] Penela D, Berruezo A, Roten L, Futyma P, Richter S, Falasconi G, Providencia R, Chun J. Cardioneuroablation for vasovagal syncope: insights on patients’ selection, centre settings, procedural workflow and endpoints-results from an European Heart Rhythm Association survey. Europace. 2024;26(5):euae106. 38781099 10.1093/europace/euae106 PMC11114473

[35] Álvarez Ortiz A, Mariño Murillo LE, Zerpa JC, Pachón CT, Pachón Mateos JC, Álvarez Mariño MA, Lopez-Lopez JP. Cardioneuroablation in the management of neurocardiogenic syncope with extracranial validation: efficacy and effects on the regulation of the cardiac autonomic system – a combined retrospective–prospective cohort study. J Interv Card Electrophysiol. 2026. 10.1007/s10840-025-02224-3. 41504825

